# The Formulated Microbicide RC-101 Was Safe and Antivirally Active Following Intravaginal Application in Pigtailed Macaques

**DOI:** 10.1371/journal.pone.0015111

**Published:** 2010-11-29

**Authors:** Alexander M. Cole, Dorothy L. Patton, Lisa C. Rohan, Amy L. Cole, Yvonne Cosgrove-Sweeney, Nicole A. Rogers, Deena Ratner, Alexandra B. Sassi, Carol Lackman-Smith, Patrick Tarwater, Bharat Ramratnam, Piotr Ruchala, Robert I. Lehrer, Alan J. Waring, Phalguni Gupta

**Affiliations:** 1 Department of Molecular Biology and Microbiology, Burnett School of Biomedical Sciences, College of Medicine, University of Central Florida, Orlando, Florida, United States of America; 2 Department of Obstetrics and Gynecology, School of Medicine, University of Washington, Seattle, Washington, United States of America; 3 Magee-Womens Research Institute and the Department of Pharmaceutical Sciences, School of Pharmacy, University of Pittsburgh, Pittsburgh, Pennsylvania, United States of America; 4 Department of Infectious Diseases and Microbiology, School of Public Health, University of Pittsburgh, Pittsburgh, Pennsylvania, United States of America; 5 Department of Infectious Disease Research, Southern Research Institute, Frederick, Maryland, United States of America; 6 Department of Biostatistics, Texas Tech University Health Sciences Center, El Paso, Texas, United States of America; 7 Division of Biology and Medicine, Brown University, Providence, Rhode Island, United States of America; 8 Department of Medicine, David Geffen School of Medicine, University of California Los Angeles, Los Angeles, California, United States of America; University of California San Francisco, United States of America

## Abstract

**Background:**

RC-101 is a congener of the antiretroviral peptide retrocyclin, which we and others have reported is active against clinical HIV-1 isolates from all major clades, does not hemagglutinate, and is non-toxic and non-inflammatory in cervicovaginal cell culture. Herein, film-formulated RC-101 was assessed for its antiviral activity *in vitro*, safety *in vivo*, retention in the cervix and vagina, and ability to remain active against HIV-1 and SHIV after intravaginal application in macaques.

**Methodology/Principal Findings:**

RC-101 was formulated as a quick-dissolving film (2000 µg/film), retained complete activity *in vitro* as compared to unformulated peptide, and was applied intravaginally in six pigtailed macaques daily for four days. At one and four days following the final application, the presence of RC-101 was assessed in peripheral blood, cervicovaginal lavage, cytobrushed cervicovaginal cells, and biopsied cervical and vaginal tissues by quantitative western blots. One day following the last film application, cervical biopsies from RC-101-exposed and placebo-controlled macaques were collected and were subjected to challenge with RT-SHIV in an *ex vivo* organ culture model. RC-101 peptide was detected primarily in the cytobrush and biopsied cervical and vaginal tissues, with little to no peptide detected in lavage samples, suggesting that the peptide was associated with the cervicovaginal epithelia. RC-101 remained in the tissues and cytobrush samples up to four days post-application, yet was not detected in any sera or plasma samples. RC-101, extracted from cytobrushes obtained one day post-application, remained active against HIV-1 BaL. Importantly, cervical biopsies from RC-101-treated animals reduced RT-SHIV replication in *ex vivo* organ culture as compared to placebo-treated animals.

**Conclusions/Significance:**

Formulated RC-101 was stable *in vivo* and was retained in the mucosa. The presence of antivirally active RC-101 after five days *in vivo* suggests that RC-101 would be an important molecule to develop further as a topical microbicide to prevent HIV-1 transmission.

## Introduction

Mucosal surfaces are the portals for sexual transmission of HIV-1 and therefore play a major role in the pathogenesis of primary infection. Antimicrobial peptides are effector molecules that constitute the first line of host defense at mucosal surfaces. Human cervicovaginal mucosa expresses a number of peptides that are intrinsically active against bacteria, fungi, and viruses such as HIV-1. However, despite their protective function, which likely contributes to the low incidence of HIV-1 transmission in women (approximately 3 in 1000 coital acts; [1], [2]), the barrier is not complete and therefore exogenously administered preventatives are necessary to reduce the incidence of HIV-1 infection.

Retrocyclins are cationic, β-sheet, 18-residue peptides, which are stable and can resist boiling, acidic conditions, and other harsh environments. Our group first reported that the rhesus homologs of retrocyclins prevent infection by both X4- and R5-strains of HIV-1 in cell culture [3]. However, whereas other antimicrobial peptides are produced by both human and non-human primates [4], humans do not naturally produce retrocyclins due to a premature stop codon that prevents translation [3], [5]. The remainder of the retrocyclin gene is remarkably intact, and thus we used the genetic information contained therein to recreate retrocyclin synthetically [3]. We determined that retrocyclin potently protected primary PBMCs and CD4^+^-enriched T cells from *in vitro* infection by both X4 and R5 strains of HIV-1 [3], and exhibited much greater activity than other human antimicrobial peptides tested [6].

From a screen of over 120 synthetic analogs of retrocyclin, we identified one called “RC-101” that exhibited a number of beneficial properties that indicate suitability for further development as a topical microbicide. RC-101 inhibited the formation of HIV-1 proviral DNA [3], [7], and exhibited broad activity against many clinical isolates of HIV-1 from all major subtypes [8], [9]. Retrocyclins such as RC-101 prevent HIV-1 entry by binding to the heptad repeat 2 (HR2) region of HIV-1 gp41, which precludes six-helix bundle (6HB) formation and subsequent viral fusion [10]–[12]. Furthermore, RC-101 effectively inhibits HIV-1 infection of organotypic cervicovaginal tissues without inducing toxicity or inflammation [7], and induces only minimal resistance in HIV-1 that can be overcome with slight increases in peptide concentration [10]. Together, these properties suggest that RC-101 would be an ideal molecule to develop as a topical microbicide to prevent HIV-1 transmission.

An ideal topical microbicide product should be effective in preventing HIV infections while not irritating the mucosal surface or adversely affecting normal flora of the vagina after chronic usage. In order to protect oneself during each sexual encounter, women will require repeat applications of the microbicidal formulation. For this reason it is imperative that studies of multiple perturbations with candidate microbicides be performed. Because clinical trials are too cumbersome and expensive for screening purposes, animal models are desirable. The pigtailed macaque model is a well-established system to evaluate microbicide agents as it has several advantages over small animals [13], [14]. The reproductive tract of the pigtailed macaque is similar to that of the human: it has regular menstrual cycles of 28 to 30 days and exhibits typical hormonal and genital tract changes as compared to women. Moreover, the vaginal flora is remarkably similar to the human, including the presence of endogenous lactobacilli, and in particular the beneficial H_2_O_2_-producing lactobacilli [15], [16]. The pigtailed macaque model has been used to evaluate several microbicidal compounds to determine whether repeated use of these compounds would have deleterious effects on the vaginal environment [13], [14], [17]–[22]. Given these beneficial metrics in assessing topical microbicide safety, we chose to utilize the pigtailed macaque model to evaluate RC-101 *in vivo* [23].

In this report, we evaluated a quick-dissolving film of RC-101 applied daily for four days to pigtailed macaques, for its safety *in vivo*, bioavailability, and antiviral activity. Our data indicate that film-formulated RC-101 was safe and retained in the cervical and vaginal tissues. Importantly, cervical biopsies from RC-101-instilled animals reduced RT-SHIV replication in an *ex vivo* organ culture model.

## Results and Discussion

### Activity of formulated RC-101 is equivalent to unformulated peptide

**Figure 1:**
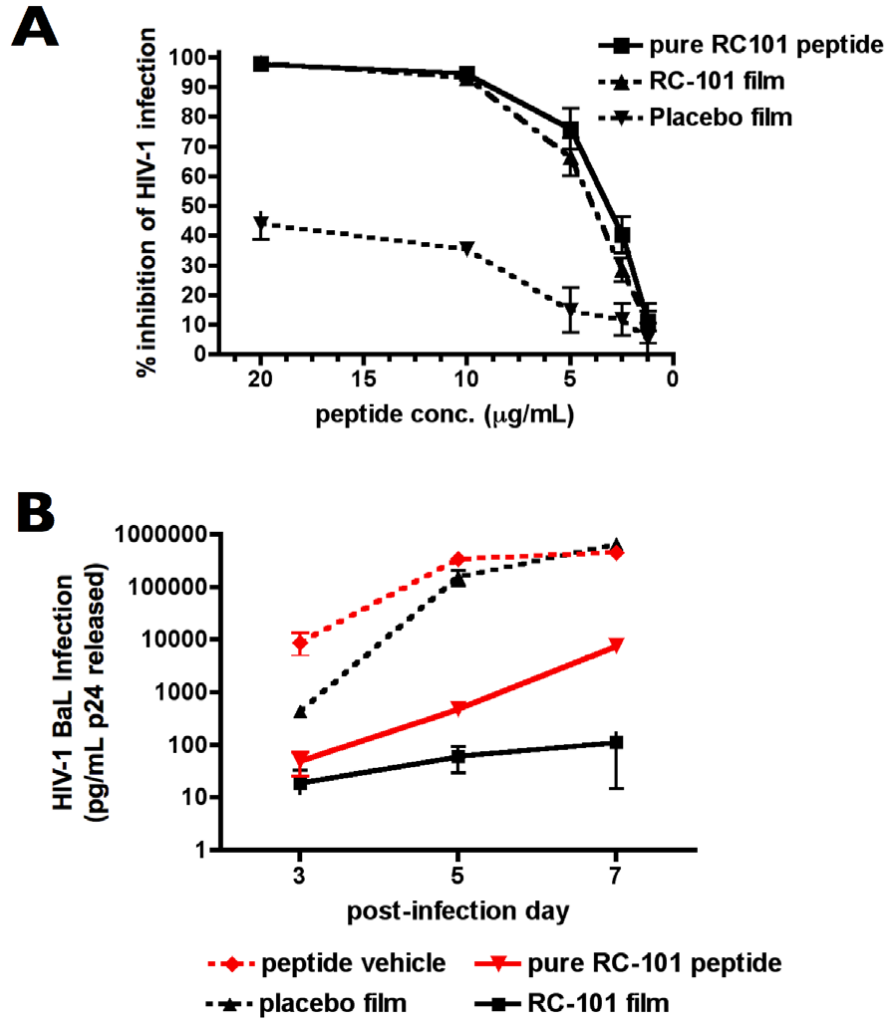
Activity of formulated RC-101 is equivalent to unformulated peptide. Antiviral activities of RC-101-containing films and placebo films against HIV-1 strain BaL were compared against unformulated “pure” RC-101 peptide in TZM-bl reporter assays expressed as percent inhibition of HIV-1 infection (**A**) and p24^gag^ release assay in PM1 cells expressed in pg/ml of p24 released (**B**). p24^gag^ differed between formulated and unformulated RC-101 at days 5 and 7, *P*<0.001 and *P* = 0.019, respectively. Error bars represent SEM; n = 2-5.

We previously reported that unformulated RC-101 was active against multiple clinical isolates of HIV-1 from all subtypes, and was neither toxic nor proinflammatory to cervicovaginal cells or tissues both *in vitro* and *ex vivo* [7]. In the current study, we evaluated RC-101 that was formulated in a quick-dissolving film suitable for *in vivo* intravaginal delivery in pigtailed macaques. We first assessed whether RC-101 retained antiviral activity following formulation. Film-formulated RC-101 (2000 µg/film) and placebo control films both rapidly dissolved in culture media (<5 min), and upon dissolution completely released RC-101 into the media as measured by quantitative immuno-dotblots (data not shown). RC-101 and placebo films were then evaluated *in vitro* against the R5-tropic HIV-1 strain BaL in TZM-bl reporter assays (**Figure 1A**; n = 5), which quantified infection as a measure of Tat-driven luciferase production, and PM1 assays (**Figure 1B**; n = 3–5), which measured infection by quantifying viral p24^gag^ antigen release in the supernatants of these lymphocytic cells. In both assays, film-formulated RC-101 exhibited potent antiviral activity, which was equivalent to unformulated RC-101 tested at the same concentration. At 5–10 µg/ml, film-formulated RC-101 inhibited more than 90% of HIV-1 replication. Interestingly, placebo films exhibited modest anti-HIV-1 activity in both assays. Similarly, by measuring p24 release in culture supernatant, the activity of RC-101 films was significantly higher at days 5 and 7 than unformulated RC-101 (**Figure 1B**; n = 2-5; *P*<0.001 and *P* = 0.019, respectively). Collectively, these results suggest that film-formulated RC-101 retained its potent antiviral activity. Furthermore, excipients within the films do not adversely affect antiviral activity, and instead might serve to augment antiviral activity of RC-101.

### RC-101 was safe and well tolerated when applied intravaginally in pigtailed macaques

Evaluation of safety of RC-101 was conducted *in vivo* in a pigtailed macaque model using films that each contained 2000 µg RC-101. This dose was selected for two reasons: First, evaluation of safety of a product *in vivo* should be conducted at higher doses to identify more accurately any toxic effects of the product. Second, it is likely that doses required for efficacy *in vivo* are several fold higher than doses required for efficacy *in vitro*. Indeed, the dose chosen for the *in vivo* studies herein was 1000 times higher than the IC_50_ of RC-101 *in vitro* [8].

**Figure 2:**
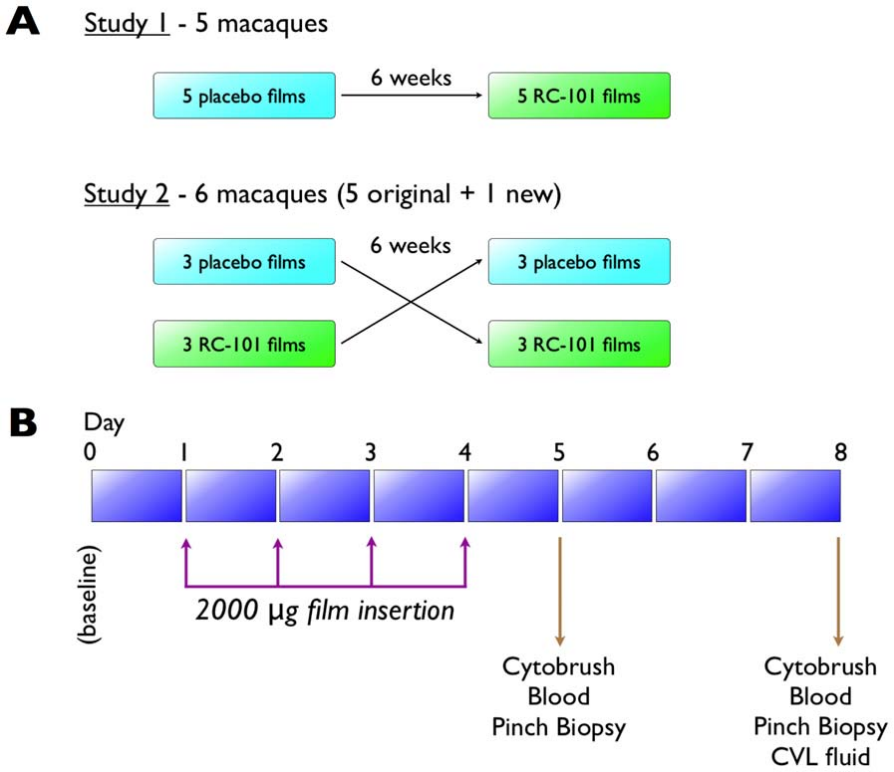
*In vivo* study protocol in pigtailed macaques. (**A**) Two studies were performed. The first included five macaques, in which each monkey received placebo films followed six weeks later with RC-101 films. The second study included the five original macaques plus an additional monkey, in which half received RC-101 films and half received placebo films. Six weeks later, each monkey receiving the opposite film type. (**B**) Baseline measurements for colposcopy, vaginal pH, and microflora were obtained at day 0. At days 1–4, RC-101- or placebo-containing films were instilled intravaginally. At days 5 and 8, cytobrushes, blood, and pinch biopsies were obtained, and at day 8 CVL fluids were obtained from each monkey.

**Figure 3:**
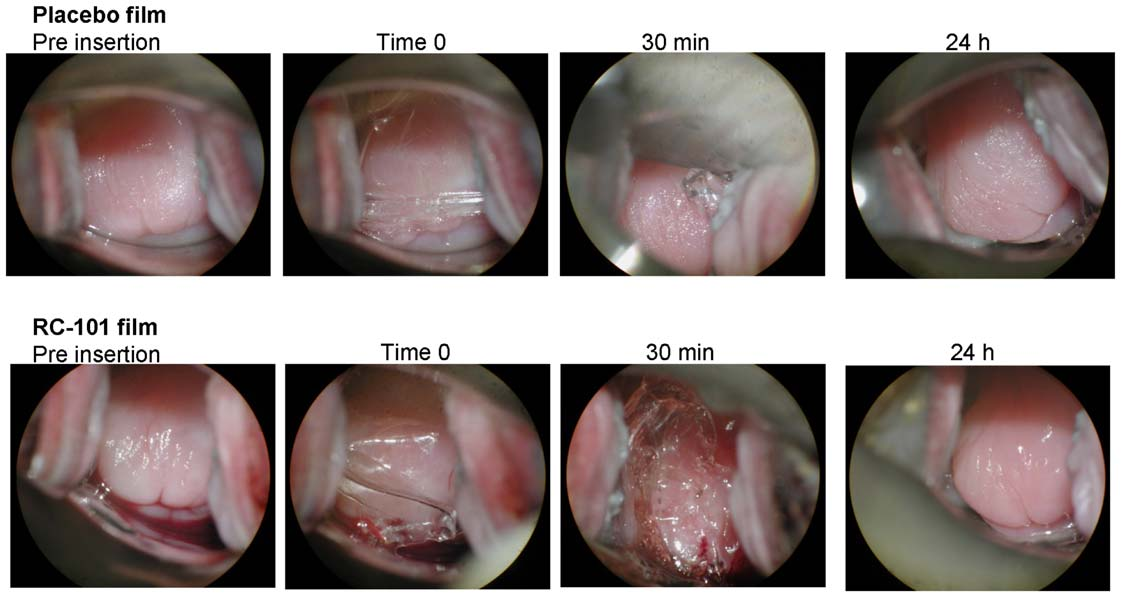
Colposcopy of the cervicovagina reveals no adverse effects of RC-101 films. Images from colposcopic examination of cervicovaginal mucosa of a representative pigtailed macaque obtained prior to film insertion, at the time of insertion (Time 0), and 30 min and 24 hr after film insertion. Note the absence of mucosal aberrations at all time points with either RC-101 films or placebo films.

*In vivo* safety evaluations were conducted as described in **Figure 2**, and colposcopy, pH and vaginal microflora determinations were the parameters used to evaluate safety of RC-101 films. The colposcopy of the cervix of representative animals before RC-101 or placebo film insertion, at the time of insertion, and 30 min and 24 hr after film insertion is shown in **Figure 3**. The film was gelatinous by 30 minutes and by 24 hrs complete disintegration of the film was observed. No visual changes were seen in the cervix following RC-101 or placebo film exposure. The visual observation was repeated after each film application (total of four films per animal over four consecutive days) and no visual changes were observed in any of the colposcopic evaluations, suggesting RC-101 was safe.

**Figure 4:**
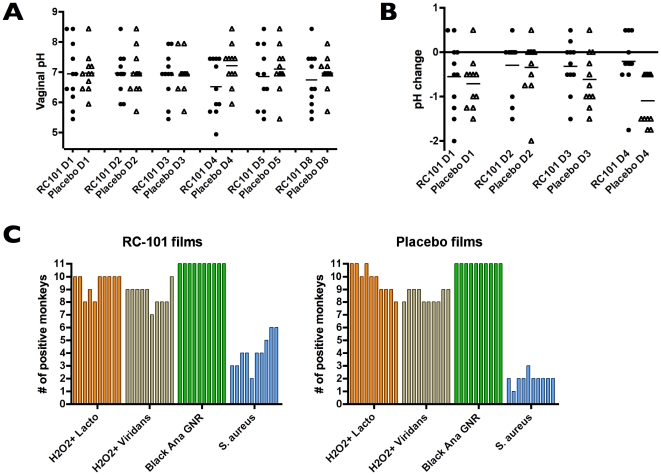
RC-101 films do not alter vaginal pH or microbiological profile of the cervicovagina. (**A**) Vaginal pH was measured prior to film instillation at days 1-4 (D1-D4) and at followup (D5 and D8). Each measurement represents one experimental condition in a pigtailed macaque. Horizontal lines represent the mean value for all 11 replicates. No differences were observed between monkeys that were receiving RC-101 films or placebo films. (**B**) represents the difference in vaginal pH between the time zero condition and 30 minutes after film instillation for each day films were applied (D1–D4). Horizontal lines represent the mean value for all 11 replicates. A significant difference was observed at Day 4 (*P* = 0.003), with the placebo films inducing a greater change in vaginal pH than the RC-101 films. (**C**) Four (of twenty) microbes are presented for both RC-101 films and placebo films. For each microbe, there are 10 vertical bars, representing the number of replicates (from a total of 11) that were positive for the microbe indicated. The ten bars for each microbe signify the following from left to right: Day 1 (D1) time zero (t0), D1, time 30 min (t30), D2 t0, D2 t30, D3 t0, D3 t30, D4 t0, D4 t30, D5 and D8.

Vaginal pH was monitored 30 min after each film application and at days 5 and 8 after the last film application (**Figure 4A and 4B**). No significant changes in pH were observed after application of RC-101 films, with the pH remaining within the normal range of macaque vaginal pH for all films tested. Interestingly, thirty minutes following application at Day 4, placebo films reduced vaginal pH as compared to RC-101 films (**Figure 4B**; *P* = 0.003). The finding that RC-101 films did not alter vaginal pH at any time point exemplifies the safety of this formulated compound. Vaginal microflora examined showed detection of H_2_O_2_-producing *Lactobacilli spp.*, H_2_O_2_-producing *Viridians* spp., *Staphylococcus aureus*, black anaerobic gram negative rods (**Figure 4C**) and 16 other microorganisms (not shown). For 19 microorganisms, no discernible differences were found between the RC-101 films and placebo films. There appeared to be a trend towards several RC-101-exposed monkeys having the presence of *S. aureus* towards the end of the study. In total, microbiological evaluation of vaginal microflora after application of RC-101 and placebo films (days 1 to 4 during film application; and, followup days 5 and 8) suggest that RC-101 had minimal effects on the microbiota of the vaginal vault. Likewise, assessment for the presence of neutrophils from Gram-stained wet mounts revealed no appreciable difference between RC-101 and placebo films (not shown). These collective findings underscore the safety of RC-101 when applied intravaginally in pigtailed macaques.

### RC-101 was retained in the cervicovagina up to four days post-application

**Figure 5:**
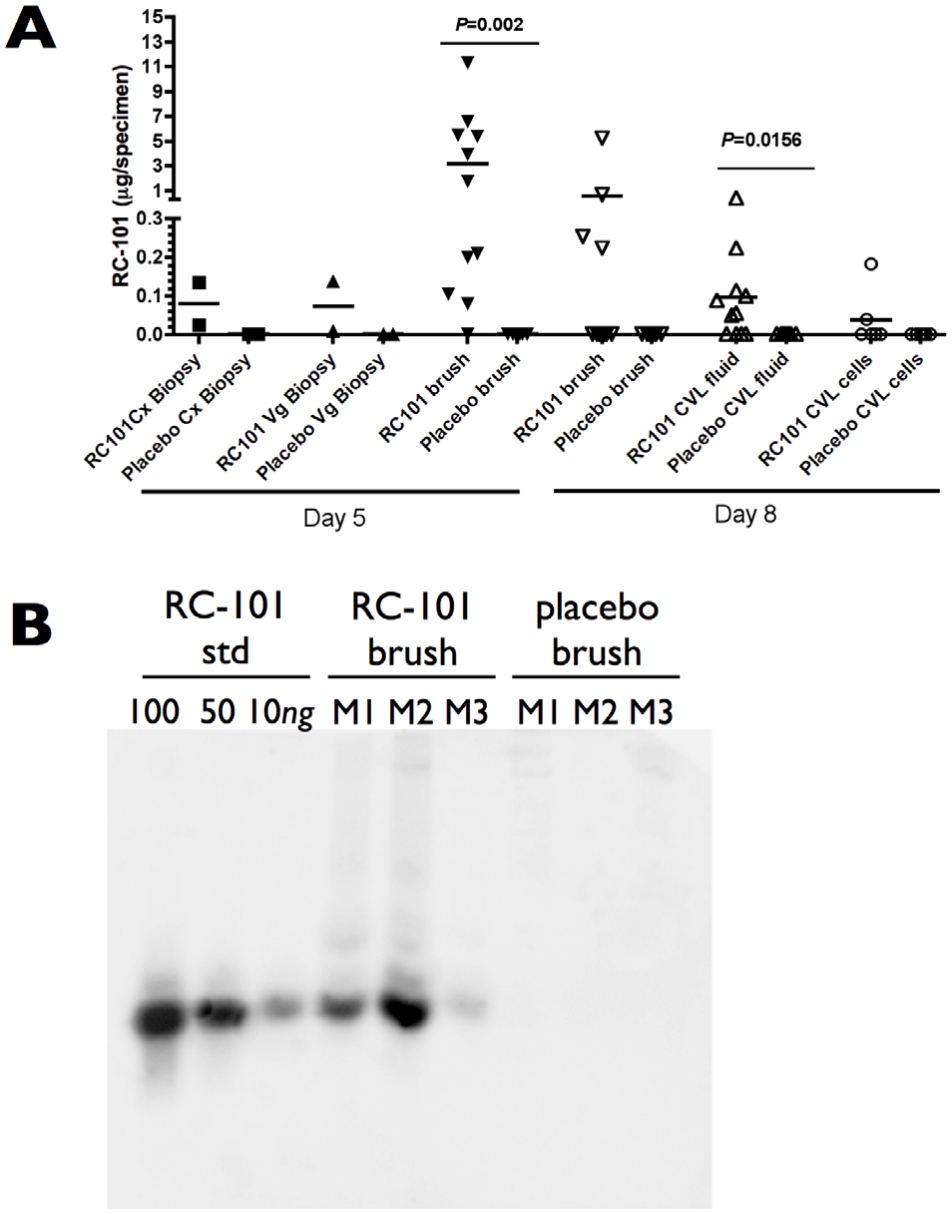
RC-101 was retained in the cervicovagina up to four days post-application. RC-101 was assessed by quantitative western blot from acetic acid-extracted samples of cervical biopsies, vaginal biopsies and cytobrushes at day 5, and cytobrushes and CVL fluid at day 8 (**A**). Note that CVL fluid was subdivided into both the cellular portion and the fluid portion. A representative western blot for RC-101 in cytobrushes at day 5 is shown in (**B**), with “M1”, “M2”, and “M3” signifying three individual monkeys. Note that the same monkey received both RC-101 films and placebo films six weeks apart, and that RC-101 was absent in cytobrushes obtained from monkeys that received placebo films. The *P*-values presented were adjusted for multiple comparisons using Tukey method.

We next assessed for the presence of RC-101 in cytobrushes, serum, plasma, and biopsies from both the cervix and vagina at days 5 and 8, as well as cervicovaginal lavage (CVL) fluid at day 8 (**Figure 5**). Each sample was extracted in 10% acetic acid to liberate RC-101, and the clarified extracts were lyophilized, resuspended in 0.1% acetic acid, and subjected to quantitative anti-RC-101 western blot analyses using synthetic unformulated RC-101 as the standard (**Figure 5B**). RC-101 was present at the highest concentrations in day 5 cytobrushes from all but one RC-101-treated monkeys, with four monkeys' cytobrushes still containing RC-101 at day 8 (**Figure 5A**). Considering how few cells are routinely recovered using the cytobrush technique, the amounts of RC-101 retained in the vaginal mucosa *in vivo* would therefore be expected to be quite high. Notably, for cytobrush samples, the concentration of RC-101 was directly proportional to the amount of protein within a given sample (data not shown), suggesting that the differences observed between samples may be attributed in part to variability in the number of cells retrieved by brushing. At day 5, RC-101 was also present in both cervical biopsies and one of two vaginal biopsies; however, due to the small size of the pinch biopsies, the amount of RC-101 recovered was near the limit of detection. CVL fluid in general contained low amounts of RC-101, further implying that RC-101 might be retained on or within the cervicovaginal epithelia. RC-101 was not detected in plasma or serum from any RC-101-treated animal, nor was RC-101 detected in any of the placebo cytobrush, biopsy, serum, plasma, or CVL samples. The finding that RC-101 was present in an intact form (evidence by Western blot) at measurable concentrations even four days after the last film application was remarkable when one considers its prolonged contact with the cervicovaginal mucosa. RC-101 may thus be suitable as a topical microbicide wherein the product could be used prophylatically in a manner dissociated from coitus.

### RC-101, extracted from cytobrush samples, remained active against HIV-1 and SHIV

**Figure 6:**
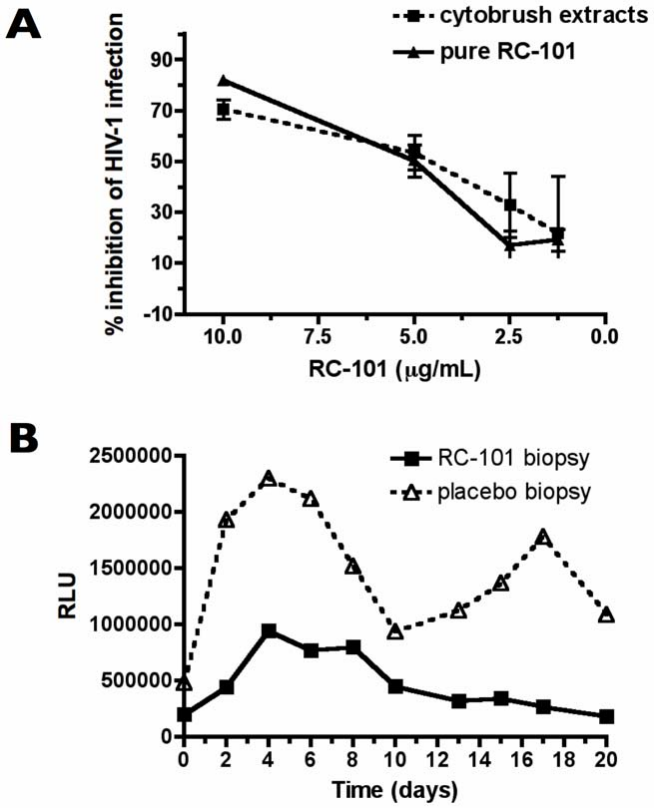
Presence of RC-101 in cytobrush samples from cervix and its antiviral activity *in vitro* and *ex vivo*. (**A**) Acetic acid-extracted day 5 cytobrush samples from three monkeys were assayed against HIV-1 strain BaL in a TZM-bl reporter assay, and compared to unformulated “pure” RC-101. No statistical differences were observed between extracted and pure RC-101 samples. (**B**) RT-SHIV replication in tissues from monkeys exposed to film formulated RC-101 or placebo film in an organ culture. The amount of virus in culture supernatant was quantified in a TZM-bl assay. Means of two tissues per condition are given.

To determine the bioavailability of RC-101 in the cervicovaginal environment after prolonged exposure, cytobrush samples were extracted and assessed for antiretroviral activity against HIV-1. RC-101 was extracted from day 5 cytobrushes (n = 3) using 10% acetic acid, and RC-101 concentration was determined by quantitative western analyses. Extracted RC-101 was assayed for anti-HIV-1 activity against HIV-1 strain BaL in TZM-bl cells (**Figure 6A**). Extracted film-formulated RC-101 exhibited antiviral activity in a dose response manner with the highest activity at 10 µg/ml.

### *C*ervical tissues, exposed to film-formulated RC-101, are resistant to *ex vivo* RT-SHIV challenge

In a final test for efficacy, we obtained four macaque cervical biopsies, two each from RC-101-treated and placebo-treated animals, and challenged each biopsy with RT-SHIV for 24 hrs in an organ culture. Viral replication was monitored by measuring infectious virus in TZM-bl cells. **Figure 6B** reveals that biopsies obtained from RC-101-treated macaques produced substantially less virus than biopsies from placebo-treated animals. Taken together, our data demonstrate that RC-101 was retained in the cervicovaginal mucosa in its active form for extended periods of time, further bolstering the utility of RC-101 as a topical microbicide. Moreover, our data indicate that *in vivo* administration of a topical microbicide followed by *ex vivo* viral challenge in both cell and tissue cultures is a highly useful approach to assess the effectiveness of microbicide candidates *prior* to expensive *in vivo* efficacy studies in nonhuman primates.

One of the unique features of this study lies in the plan to characterize and develop an ancestral human antimicrobial peptide into a topical microbicide to prevent HIV-1 infection. Phylogenetic evidence from our group indicates that human retrocyclins were silenced after the orangutan and hominid lineages had diverged [5]. Given that retrocyclin is an effective inhibitor of HIV-1 infection, the evolutionary loss of retrocyclin may have contributed to HIV-1 susceptibility in modern humans. If so, then the topical administration of retrocyclins, such as RC-101, to humans would restore natural effector molecules that were never relinquished by many of our nonhuman primate relatives. Not only might the use of a human-derived peptide reduce the toxicity and allergy that can be associated with chemically synthesized compounds or non-mammalian peptides, to our knowledge HIV has not encountered retrocyclin “in the wild” and thus far should not have developed resistance. Indeed, RC-101 itself induces little resistance in HIV-1 [10], further underscoring its potential as a topical microbicide.

In summary, based on low toxicity, good bioavailability in the cervix and vagina, and *in vitro* and *ex vivo* challenge data, RC-101 appears to be a promising compound suitable for further development as a topical vaginal microbicide to prevent sexual transmission of HIV-1.

## Materials and Methods

### Ethics statement for *in vivo* evaluations in pigtailed macaques

A total of six sexually mature female *Macaca nemestrina* were obtained from a colony of animals at the Washington National Primate Research Center. Prior approval for use of monkeys in this protocol was obtained from the Institutional Animal Care and Use Committee at the University of Washington. Animals were handled humanely, and experiments were performed within the National Institutes of Health's laboratory animal use guidelines.

### *In vivo* evaluations in pigtailed macaques

Two studies were performed as delineated in **Figure 2A**. Films were applied to the animals following the experimental design in the schema shown in **Figure 2B**. Films of RC-101 2000 µg/film were assessed for safety with vaginal application and compared to a placebo film. Each animal controlled for itself by completing both arms of the study (first RC-101 and then placebo film). For each study, each animal received one film/day for a period of 4 days. On study days 1 to 4, colposcopy assessments, and swabs for vaginal pH, neutrophil quantification, and microflora were obtained at time zero. Immediately following specimen collections, an intravaginal application of 1 film (RC-101 or placebo) was administered to each animal. Colposcopy and vaginal swabs were again performed 30 min after film application. Colposcopy was conducted every day after 30 min of application and before the next day application. On study days 5 and 8, colposcopy, and swabs for vaginal pH, neutrophils and microflora were collected to document recovery. Standardized colposcopic assessments were conducted by a team of three cross-trained individuals. In order to document the appearance of the cervicovaginal tissues prior to potential tissue perturbation caused by sampling, colposcopy took place immediately after speculum placement before any swab collections. Vaginal pH was determined by rolling a swab of vaginal secretions onto a pH indicator strip with a resolution of 0.5 pH unit. A second vaginal swab was collected and immersed in a transport tube (Port-a-Cul; Becton Dickinson Microbiology Systems, Cockeysville, MD) for semi-quantitative microbiologic characterization. Microbiologic characterization was conducted in the laboratory of Sharon Hillier, Ph.D. (Magee Womens Research Institute, Pittsburgh, PA). Neutrophils were quantified through enumeration of Gram-stained vaginal wet mounts as described previously [24]. In addition, cytobrushes, serum, plasma, and pinch biopsies at days 5 and 8 and cervicovaginal lavage at day 8 were collected, and sent to the University of Central Florida for evaluation of RC-101 concentration and anti-HIV-1 activity. Cervical biopsies were also sent to the University of Pittsburgh for *ex vivo* analyses of anti-SHIV activity. Notably, for the second study (**Figure 2A**), samples were sent to investigators at the University of Central Florida and the University of Pittsburgh in a blinded fashion.

### Extraction of RC-101 from macaque samples

Macaque samples (cervix/vagina biopsy, cytobrush, CVL, serum, or plasma) were harvested at the University of Washington, shipped on dry ice, and stored at −80°C. To extract RC-101, samples were mixed with glacial acetic acid to a final concentration of 10% (v/v), vortexed for 20 min at RT, and centrifuged at 13,000 rpm for 10 min at 4°C. Supernatant containing acid-soluble RC-101 was collected, and insoluble material was re-extracted with 5 volumes of 10% acetic acid for another 20 min. Following centrifugation, the supernatants were pooled, vacuum concentrated, resuspended to their original volume with 0.1% acetic acid/molecular grade water (v/v), and stored at −80°C prior to immuno-detection.

### RC-101 synthesis, processing, purification, and formulation as intravaginal films

The 18 amino acid RC-101 peptide was prepared on a 0.25 mmol scale with an ABI 431A peptide synthesizer using FastMoc™ chemistry [25] as described in [3]. In brief, Fmoc-Wang resin (100-200 mesh) was used to synthesize the reduced monomer (Novabiochem, San Diego, CA) and all residues were double coupled to insure optimal product yield. After cleavage and deprotection of the peptide from the resin using trifluoroacetic acid:1,2-ethanedithiol: thioanisole:water, 10∶0.25∶0.5∶0.5 (v:v), the linear reduced RC-101 was separated from the resin by filtration and precipitated from solution with tertiary butyl ether and purified by preparative scale reversed-phase (RP)-HPLC using a Vydac C18 column. The purified reduced RC-101 was oxidized with 10 mM ammonium acetate buffer pH 7.5 for 18 hrs by stirring at 25°C, followed by vacuum-concentration and separation of the oxidized from unreacted peptide with RP-HPLC [3]. Oxidized RC-101 was cyclized by dissolving at a concentration of 0.5 mg/ml in DMSO that contained 60 moles of EDC (1-ethyl-3-(3-dimethylaminopropyl) carbodiimide) and 20 moles of HOBt (N-hydroxbenzotriazole) per mole of RC-101 and stirred in the dark at 20°C for 18 hrs. The solution was concentrated at 80°C and the derivative separated from non-cyclized material by RP-HPLC. After each processing step, peptides were subjected to MALDI-TOF mass spectrometry to confirm homogeneity and that the measured mass agrees well with its expected mass. RC-101 was formulated by solvent casting techniques as a quick-dissolving polymeric 27.5 mm ×33.5 mm vaginal film, composed of 2000 µg RC-101, 6% polyvinyl alcohol (Kuraray America Inc., New York, NY), 0.12% hydroxypropyl methylcellulose 6 cps (HPMC) (Sigma, St. Louis, MO), and 3% glycerin (Dow Chemical Co., Midland, MI) as described in Sassi and colleagues [26]. All films were stored in PET/Aluminum foil pouches (Amcor Flexibles Healthcare, Inc., Mundelein, IL) until used for subsequent analyses.

### Cells and Virus

The following reagents were obtained through the NIH AIDS Research and Reference Reagent Program, Division of AIDS, NIAID, NIH: TZM-bl cells from Dr. John C. Kappes, Dr. Xiaoyun Wu and Tranzyme Inc. [27]–[30], PM1 cells from Dr. Marvin Reitz [31], and HIV-1 Ba-L from Dr. Suzanne Gartner, Dr. Mikulas Popovic, and Dr. Robert Gallo [32]. TZM-bl is a HeLa cell line stably expressing large amounts of CD4 and CCR5 and has luciferase and B-galactosidase genes under control of the HIV-1 promoter. TZM-bl cells were maintained with DMEM high glucose (Mediatech, Inc., Manassas, VA) containing 100 U/mL penicillin/0.1 mg/mL streptomycin (Mediatech) and 10% (v/v) heat-inactivated FBS (Gemini Bio-Products, West Sacramento, CA). PM1 cells readily propagate R5 and X4 HIV-1. They were maintained at 0.4–0.8×10^6^/mL in RPMI 1640 (Mediatech) containing 10 mM HEPES, 100 U/mL penicillin/0.1 mg/mL streptomycin, and 20% (v/v) FBS. HIV-1 BaL stocks were prepared by infecting 3×10^6^ PM1 cells for 3 hr, washing the cells of excess virus, and growing the cells at 0.75×10^6^/mL. Cell supernatants containing propagated virus were collected every other day starting at day 3 post-infection. Supernatants were clarified by centrifugation, filtered through at 0.45 uM nylon syringe filter, and stored at −80°C in aliquots for use as virus stocks. These HIV-1 BaL viral stocks were quantified using an HIV-1 p24 ELISA (Perkin Elmer, Freemont, CA), and virus titers (TCID_50_/mL) were determined by titration on TZM-bl and PM1 cells.

### Antiviral Assays

Placebo films and films formulated with 2000 µg RC-101 were dissolved in warm growth media so that the final presumptive concentrations administered to cells were 1.25–20 µg/mL peptide. As an initial screen for anti-viral activity, TZM-bl reporter cells were seeded at 4000 cells/well in 96W black plates (Corning, Lowell, MA) and infected the next day with 800 TCID_50_/mL (MOI = 0.02) HIV-1 BaL in the presence of 1.25–20 µg/mL of pure RC-101 peptide (or the equivalent amounts of 0.01% acetic acid vehicle) or the above mentioned dilutions of placebo versus RC-101 formulated films. After 24 hr, supernatants were removed and cells were lysed with 100 uL of 1X Glo Lysis buffer (Promega Corp., Madison, WI) and then frozen. Bright Glo luciferase assay buffer (Promega) was then added directly to the thawed lysates and luciferase activity was measured on an LMax luminometer (Molecular Devices, Sunnyvale, CA). Results were confirmed using a PM1 cell infection assay that measures propagation of HIV-1 over 7 days. Cells (1×10^5^/0.1 mL) were infected with HIV-1 BaL (1600 TCID_50_/0.1 mL, MOI = 0.02) for 3 hr in the presence of pure RC-101 or diluted films, then washed of excess virus, and left to incubate (37°C/5% CO_2_) in 0.5 mL growth media containing peptide or diluted films. On day 3 post-infection, the supernatants were collected, filtered and frozen, and the cells were resuspended in 1 mL fresh growth medium containing pure peptide or diluted placebo versus film-formulated RC-101 for two more days. On day 5 post-infection, this step was repeated. PM1 cell infection was quantified by performing HIV-1 p24 ELISA on supernatants from days 3, 5, and 7.

### Gel electrophoresis and semiquantitative western blot analysis

Standards and samples were prepared in 0.1% acetic acid containing 0.1% cetyltrimethyl ammonium bromide (CETAB) and then admixed 1∶1 with AU loading dye (9M urea in 5% acetic acid, colored with methyl green). A 12.5% native acid-urea (AU) PAGE was used to separate proteins based on their net charge density, and gels were electroblotted to PVDF in ice cold 0.7% acetic acid/10% methanol for 22 min at 180 mA as described previously (Valore EV et al. JCI 101, 1998, and Cole AM, JI 169, 2002). Membranes were fixed with 0.1% glutaraldehyde diluted in Tris-buffered saline (TBS, 20 mM Tris pH 7.5, 500 mM NaCl), blocked with Superblock (Pierce, Rockford, IL) containing 0.05% Tween20 for 30 min at 37°C with gentle agitation, and incubated on a rocker overnight with anti-RC-101 rabbit polyclonal antisera diluted 1∶1000 in 1∶3 Superblock (1 part Superblock, 2 parts TBS/0.05% Tween20). Membranes were then washed thrice with low salt TBS (20 mM Tris, 0.9% NaCl, pH 4.5) containing 0.1% BSA and 0.1% Tween20, blocked with Superblock for 15 min at 37°C, and incubated for 1 hr with goat anti-rabbit IgG-HRP (Pierce) diluted 1∶10000 in 1∶3 Superblock. Following three more washes with TBS/BSA/Tween20 and 2 washes with TBS/Tween20, blots were developed with ChemiGlow (Cell Biosciences,Inc., Santa Clara, CA) and images were documented and analyzed with Bio-Rad's ChemiDoc XRS system and QuantityOne software (Bio-Rad, Hercules, CA).

### SHIV challenge of biopsies from pigtailed macaques treated with RC-101 films

Monkey cervical issues, obtained at baseline and after treatment with film formulated RC-101, were sent on cold packs in culture medium overnight from the University of Washington, Seattle to the University of Pittsburgh. Tissues were rinsed with an antibiotic wash (100 units/ml penicillin/streptomycin, nystatin, amphotericin B), and stimulated overnight with PHA (5 µg/ml). Next day, tissues were washed with PBS, placed in a 96 well plate and infected with 250 µl (TCID 50) of RT-SHIV (NIH AIDS Research and Reference Reagent Program) overnight at 37°C. Following infection the tissues were washed three times with PBS and placed in a 48 well plate on surgifoam (Absorbable Gelatin Sponge, Johnson & Johnson) that was pre-soaked in RPMI 1640 containing IL-2, penicillin/streptomycin and 20% FBS. Five hundred microliter of the same media was added to the wells to keep biopsies in an air-liquid interface. One hundred microliter samples were collected every 2–3 days and frozen at −80°C until testing. The samples were tested for infectivity in indicator TZM-bl cells using luminescence (BetaGlo Assay System, Promega). Tissue viability was tested by an MTT assay at day 20 and all tissues were found to be viable.

### Statistical analyses

Data are presented as scattergrams with mean bars which show all data points and the group measure of central tendency. If data were measured repeatedly across time or dose the group means with standard error bars were plotted with lines connecting groups across the scale the measurements were observed. The comparisons of means between two groups (e.g., formulated versus unformulated RC-101) were conducted using the two-sample t-test with unequal variances. The comparisons of means among more than two groups were conducted using the analysis of variance method. Subsequent pair-wise comparisons (two group comparisons) were calculated to be adjusted for multiple comparisons using the Tukey method (e.g., comparison of RC-101 CVL to placebo CVL at day 8 after the comparison of treatment groups at day 8).
